# Association Between Educational Attainment and Overweight/Obesity in Eight South Asian Countries: A Systematic Review

**DOI:** 10.1177/10105395251409934

**Published:** 2026-01-06

**Authors:** Rubee Dev, Zoe O’Neill, Colleen M. Norris, Valeria Raparelli, Swarnali Sarkar, Louise Pilote

**Affiliations:** 1 School of Nursing, Faculty of Applied Science, The University of British Columbia, Vancouver, BC, Canada; 2 Division of Internal Medicine, University of Ottawa, Ottawa, ON, Canada; 3 Faculty of Nursing, University of Alberta, Edmonton, AB, Canada; 4 Department of Translational and Precision Medicine, Sapienza-University of Rome, Rome, Italy; 5 Ernst and Young LLP, New Delhi, India; 6 Divisions of General Internal Medicine and Clinical Epidemiology, Department of Medicine, McGill University, Montreal, QC, Canada; 7 Centre for Outcomes Research and Evaluation, McGill University Health Centre Research Institute, Montreal, QC, Canada

**Keywords:** education, overweight, obesity, South Asia, sex, gender

## Abstract

The prevalence of overweight/obesity (OW/OB-defined by body mass index) in low- and middle-income countries is rising, and the sociodemographic characteristics of the most affected populations are changing. The relationship between education, widely recognized as a gender-related variable, and OW/OB in high-income countries is well understood; however, the impact in South Asian (SA) countries is less clear. This systematic review interrogated the relationship between educational attainment and OW/OB, by searching Ovid MEDLINE, CINAHL, EMBASE, and Web of Science for studies published after 2013, reporting the prevalence of OW/OB by educational attainment in eight SA countries. Data were extracted and the association between education and OW/OB was coded as direct, indirect, null, or U-shaped. A total of 32 studies were included in the review. The mean age was 38.5 years. The prevalence of OW/OB ranged between 4.6% and 64.4%. Females were reported to be at higher risk of OW/OB compared with males. Most of the studies reported women with higher education at greater risk of being OW/OB. SA countries are undergoing substantial transformations in their economic and social frameworks that influence how sex and gender mediate cardiovascular risk factors like OW/OB. Preventive strategies must be tailored to the unique characteristics of the SA population.

## What We Already Know

- Educational attainment is associated with overweight/obesity.
- Higher educational attainment is generally associated with lower rates of overweight/obesity.
- The relationship can be reversed in low- and middle-income countries where higher educational attainment often correlates with higher rates of overweight/obesity.

## What This Article Adds

- There is a complex interplay between sex and gender, educational attainment, and overweight/obesity.
- Women with higher education are reported to be at higher risk of being overweight/obese.
- The relationship between education and obesity can vary by sex and gender, and across different countries, cultures, and socioeconomic levels.

## Introduction

As a result of an ongoing epidemiological transition, rapid urbanization, and economic shifts, there is an increase in the burden of cardiovascular disease (CVD) risk factors in low- and middle-income countries (LMICs),^1-3^ especially in South Asian (SA) countries. South Asia has the highest global rates of CVD, with ischemic heart disease and stroke as leading causes of death.^4^ The global prevalence of obesity, which is one of the CVD risk factors, is expected to rise from 14% in 2020 to 24% by 2035.^5^ Notably, the prevalence of overweight and obesity (OW/OB) in SA countries is rising sharply, and the sociodemographic characteristics of the most affected populations are shifting in notable ways. OW/OB is strongly associated with hypertension, diabetes, dyslipidemia, and CVD, often at lower BMIs in South Asians.^6^ OW/OB are conditions defined by excessive fat accumulation, posing health risks, and are assessed using body mass index (BMI).^7^ The magnitude of the obesity epidemic in SA countries was reviewed by Jayawardena et al^8^ who reported obesity prevalence ranging from 9% to 38% among males and 4% to 48% among females. The prevalence of overweight was reported as 22% to 61% in males and 9% to 66% in females, with large variation between countries.

Lower educational attainment has long been associated with a higher risk of obesity in high-income countries (HICs) such as Canada and the United States. However, the conclusions of studies interrogating this relationship in LMICs, including South Asia, is more complex and sometimes reversed. While some studies have historically reported that an inverse relationship between educational attainment and obesity exists such that the most educated are at the lowest risk of being obese,^9^ more recent data seems to suggest that this relationship is mutable and country dependent.

Educational attainment is also widely recognized as a gendered variable. Gender refers to “the array of socially constructed roles and relationships, personality traits, attitudes, behaviours, values, relative power, and influence that society ascribes to women and men on a differential basis.”^10^ The impact of educational attainment on obesity in South Asian populations is increasingly recognized as gender-sensitive, with emerging evidence showing distinct patterns for women and men. In South Asia, gender norms (e.g., women eating last, limited autonomy in food choices) is believed to undermine the protective effects of education.^11^ Yet, no studies have examined this association. A recent study across 10 Asian countries found that women with secondary or higher education living in wealthier households had lower odds of obesity (OR = 0.71, 95% CI: [0.66, 0.76]).^12^ However, this was not specific to SA countries, was not a systematic review, and gender norms remained underexplored in this study. A previous review by Cohen et al^13^ across 91 countries found that in HICs, higher education is inversely associated with obesity, and in LMICs, higher education may be positively associated with obesity, especially among women. However, South Asian countries were underrepresented in this review, and the review did not examine the impact of gender.^13^

To the best of our knowledge, there is no dedicated systematic review that focuses exclusively on SA countries, has examined educational attainment as a primary predictor of OW/OB in South Asia, and has examined the gendered impact of education on OW/OB in South Asia. Even in Cohen’s study, only a few countries from SA were included in the review. Furthermore, a substantial body of research examining this association has been published since Cohen’s review in 2013 that needs to be updated. Hence, to interrogate the role of gender (which this study distinguishes from biological sex) in obesity as a cardiovascular risk factor in South Asia, this study systematically reviewed the literature to examine the relationship of education and obesity in eight SA countries (Nepal, India, Bangladesh, Afghanistan, Pakistan, Bhutan, Sri Lanka, and Maldives). The main objective of this article was to determine the impact of individual educational attainment on OW/OB in eight SA countries. This review will shed light on the changing relationship of educational attainment and obesity in SA countries and demonstrate the risk of grouping all of them together in analysis.

## Methods

### Search Strategy

In this systematic review, Ovid MEDLINE, CINAHL and EMBASE were searched for studies published after 2013 using a combination of Medical Subject Headings (MESH) or key search terms major for education and obesity. Studies published after 2013 was used to provide an update on the earlier review conducted by Cohen.^13^ The literature search strategy was based on previous systematic reviews and meta-analyses.^13^ A medical librarian at the McGill University was consulted to develop the search strategy and refine keywords relevant to overweight, obesity, sex, gender, and South Asia. The reference lists of included articles were manually searched for additional studies. In order to ensure that our search captured all relevant articles, country/region was not included in the search terms. Instead, abstracts and titles were screened manually to indicate where the study was completed.

Rayaan, a web-based tool was used to complete the initial screening of abstracts in a semi-automated fashion.^14^ The Preferred Reporting Items for Systematic Reviews and Meta-Analyses (PRISMA) guidelines for systematic review were followed. The protocol was registered with the International Prospective Register of Systematic Reviews (PROSPERO ID: CRD42021272174).

### Inclusion and Exclusion Criteria

**Figure 1.**
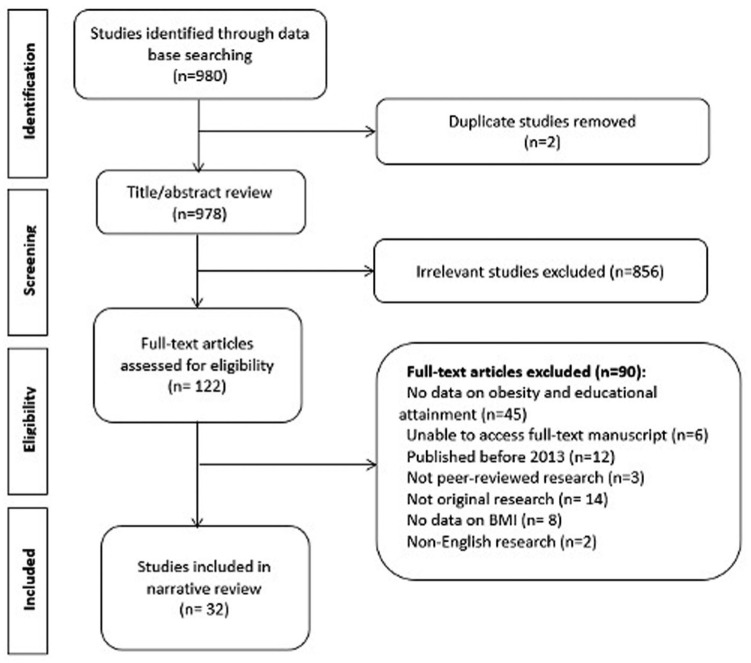
PRISMA flowchart of the systematic review literature search illustrating the identification of included studies.

Studies were included if they matched the following criteria: (1) published in English only, (2) represented original peer-reviewed research articles (not meta-analysis, review article, conference proceedings, case report/series), (3) the study took place in one of eight SA countries (Nepal, India, Bangladesh, Afghanistan, Pakistan, Bhutan, Sri Lanka, and Maldives), (4) reported an individual’s own educational status (as opposed to parent’s education), (5) reported an association or prevalence of OW/OB by educational attainment, (6) reported variability in educational attainment and obesity within the study population (eg, studies where all subjects were overweight were not included), and (7) study participants were 18 years or older (Figure 1). There were no restrictions on study design or publishing date. Studies were excluded if (1) full-text articles could not be obtained, (2) education was recognized as an effect modifier or confounder, but the relationship between education and OW/OB was not quantified, and (3) the study was a duplicate.

### Data Extraction and Quality Assessment

Rayyan, a free web-based tool for systematic review, was used for this review.^15^ Data was extracted using a standardized Garrard Matrix form.^16^ Two researchers (ZRO and RD) independently screened titles and abstracts of all articles retrieved during the initial database search. Studies that met initial inclusion criteria were subsequently screened by full-text review according to the eligibility criteria by the same two authors. Any disagreement was resolved by a third reviewer (LP). Extracted data included: the article and study characteristics, demographics of participants, clinical characteristics, gender-related variables, and cardiovascular outcomes. Studies were evaluated using the Newcastle-Ottawa Scale, which took into account biases in selection, detection, reporting, attrition risk, and confounding factors.^17^

When papers reported several effects estimates for the same population, this study abstracted the analysis that most closely estimated direct effects and adjusted for the largest number of covariates. OW/OB measured either by the global or Asian BMI categories was the preferred outcome if several measures of obesity were provided, but all measures of obesity and adiposity were acceptable. Articles often reported effect estimates for several different study populations. If the paper stratified its results by year and/or country, this study considered a “study population” to be year-specific and/or country-specific. These articles were split into as many data points as there were study populations. Current study also stratified associations reported by year based on data collection years reported in the study for our own analysis where possible to observe temporal trends.

### Definitions of Overweight, Obesity, and Educational Attainment

According to the World Health Organization classification, overweight is defined as a BMI between 25 and 29.9 kg/m^2^ and obesity is a BMI of ≥30 kg/m^2^.^7,18^ While according to the WHO cutoffs for Asian populations, overweight is defined as a BMI between 23 and 27.5 kg/m^2^ and obesity is a BMI of ≥27.5 kg/m^2^.^18,19^ Of the 32 studies included in this review, twenty studies^20-37^ used the global WHO cutoffs and nine studies^38-47^ used Asian cutoffs. Two studies^48,49^ reported data using both global and Asian cutoffs. Fifteen studies^22^,^24-26^,^28,29^,^34-36^,^39-41^,^48,50,51^ combined overweight and obese individuals for statistical analysis when reporting prevalence of OB/OW or association with educational attainment. Eleven studies^21,27,32,33,38,42,43,46,47,49^ reported them separately. Five studies^23,31,37,44,45^ only reported either obesity or overweight by educational attainment. One study^30^ did not categorize participants, and instead reported the association between BMI and educational attainment, expressed as the β-coefficient.

The categories for educational attainment varied and reflected the differences in educational systems across countries. The majority of studies created four categories for educational attainment (illiterate/no formal education, primary school, secondary/high school, and graduate school or higher).^20-22^,^24,25,28,29,35,38,40,41,43,44^,^48-52^ While other several studies categorized education into three^23,26,30,32,34,46^ or five or more categories,^27,33,36,37,39,42,45^ one study^31^ used illiterate and literate to classify educational attainment.

For the purpose of this study, the relationships between education and obesity were manually coded as *directly associated* (as education increases, so does the odds or risk of OW/OB), *inversely associated* (as education decreases, the odds or risk of OW/OB increases), *null* (no statistically significant relationship observed between education and OW/OB; *P* < .05), or *U-shaped* (if there appeared to be a curved relationship to the data).

## Results

### Study Characteristics

Among 978 studies identified, 122 were selected for full-text review and 32 met inclusion criteria (Figure 1). This included 14 studies from India for which the study data collection period spanned from 2013-2019 and included over 1.25 million participants.^20-27^,^38-42^,^50^ Six studies from Nepal were included in the review with data collected between 2013 and 2019 from a total of 29 044 participants.^28,35-37,43,48^ Four studies from Bangladesh spanning 2014-2017 with a total of 30 385 individuals were included.^29,44-46^ Furthermore, three studies from Afghanistan were included in the review from 2013 to 2018 and included 20 547 participants.^30,32,53^ Two studies from Sri Lanka from 2013 and 2019 were included in the analysis with data from 2932 participants.^49,52^ Only one study each from the Maldives (2016, n = 6634),^51^ Pakistan (2013, n = 7366),^33^ and Bhutan (2014, n = 2822)^34^ were included in this study (Table 1). Eighteen of thirty-two studies were cross-sectional by design, two utilized random sampling methods and one was prospective. A total of 1 419 825 adults represented the study population in this review, of which females comprised 87.9% and males comprised 12.1% of the population. Among the studies that reported age, the mean age of study population was 38.5 years. Risk of bias scores for all the individual studies included in the review is presented in Table 1.

**Table 1.** Characteristics of Included Studies on Educational Attainment and Overweight/Obesity (n = 32).

Study	Survey year(s)	Mean age at obesity assessment (years)/age range	Country	Study design	Sample size	OB/OW defined according to global or Asian BMI categories	Main findings related to the review	Prevalence of OB/OW	Risk of bias score
Agarwal et al^20^	2013-2014	Mean age: 42.7 years	India	Cross-sectional study	38457 (men: 43%, women: 57%)	Global	Obesity highest in participants with graduate school and above education (aOR: 2.3, 95% CI: [1.8, 2.9]).	OW: 17.4% OB: 4.6%	5
Sharma et al^21^	2013	Mean age: 52 years	India	Cross-sectional study	1214	Global	Abdominal as well as generalized obesity high among men and women in high and medium educational status groups.	OW/OB: 64.0%	5
Gupta et al^22^	2015	Range: ≥ 60 years	India	Cross-sectional study	981	Global	High prevalence of OW/OB observed among participants with high school education or above (aOR: 1.9, 95% CI: [1.1, 3.5]).	OW: 18.0% OB: 4.6%	9
Gupta et al^38^	2015	Range: 15-49 years	India	Cross-sectional study	644006 women	Asian	The prevalence of OW/OB increased with higher educational attainment.	OW: 22.7% OB: 10.8%	9
Selvaraj et al^39^	2015	Range: ≥ 18 years	India	Cross-sectional study	2399	Asian	Adults who had literacy beyond primary level were around two times more likely to have clustered NCD risk factors (aOR: 1.8, 95% CI: [1.1, 5.5]) compared with illiterates. However, such association was not found among adults who studied beyond higher secondary level.	OW/OB: 64.4%	7
Basu et al^23^	2016-2017	Mean age: 38.7 years	India	Cross-sectional study	103 women	Global	Middle/Secondary/Higher education associated with higher OW (OR: 1.9, 95% CI: [0.6, 7.5]) compared with Illiterate/Primary education.	OW: 44.7% OB: 13.6%	6
Luhar et al^24^	2016	Range: 15-54 years	India	Cross-sectional study	722413	Global	The prevalence of OW/OB was highest among participants with higher education, whereas the lowest prevalence of OW/OB was found among participants with no education.	OW/OB rural male: 14.0% OW/OB rural female: 17.0% OW/OB urban male: 26.0% OW/OB urban female: 36.0%	10
Chowdhury et al^50^	2017	Mean age: 24.1 years Range: 18-30 years	India	Cross-sectional study	2000	Global	The prevalence of OW/OB was higher among participants with higher than secondary education compared with those with no education (aOR: 1.1, 95% CI: [0.6, 1.9])	OW: 20.6% OB: 5.7%	10
Kokane et al^25^	2017	Mean age: 40.4 years 18-69 years	India	Cross-sectional study	5680	Global	Higher education levels increased the risk of being overweight (OR: 1.6, 95% CI: [1.3, 2.0])	OW/OB: 18.7%	9
Rai et al^40^	2017	Range: ≥ 18 years	India	Prospective cohort study	24115 (men: 10915, women: 13200)	Asian	Individuals with educational attainment of grade 6 or higher were more likely to be OW compared with individuals who were illiterate, and the association tended to be stronger among men (aOR: 3.3, 95% CI: [2.5, 4.3]) than women (aOR: 1.9, 95% CI: [1.5, 2.6]).	OW/OB in men: 17.3% OW/OB in women: 24.7%	9
Singhania et al^26^	2018-2019	Mean age: 53.7 years Range: 40-60 years	India	Cross-sectional survey	400 women	Asian	Women with higher education were at higher risk of being OW/OB than women with no formal education (aOR: 2.3, 95% CI: [1.2, 4.2]).	OW: 26.0% OB: 9.5%	9
Sarveswaran et al^41^	2019	Mean age: 39.7 years Range: 18-69 years	India	Cross-sectional survey	1234	Asian	Obesity significantly associated with lover levels of education compared with participants with higher secondary and above education (aOR: 1.26, 95% CI: [1.1, 1.5]).	OW: 36.3%	7
Sivanantham et al^42^	2019	Mean age: 44.3 years Range: 18-69 years	India	Cross sectional survey	2415 (men: 1086, women: 1329)	Asian	Participants with graduate and above education were at higher risk of being OW/OB compared with participants with no formal education (OR: 1.2, 95% CI: [1.1, 1.3]).	OW: 17.6% OB: 46.1%	10
Rai et al^27^	NA	Range: 20-60 years	India	Random Sampling method	657 women	Global	The prevalence of OW/OB was highest among those women who had attained secondary education followed by primary education.	OW: 30.4% OB: 15.1%	4
Aryal et al^28^	2013	Range: 15-69 years	Nepal	Cross-sectional survey	4200 (men: 32%, women: 68%)	Global	Participants with higher education were at higher risk of being OW/OB compared with participants with no formal education (OR: 1.9, 95% CI: [1.5, 2.3]).	OW/OB male: 21.0% OW/OB female: 21.8%	10
Dhungana et al^37^	2014-2015	Mean age: 42.5 years Range: 18-70 years	Nepal	Cross-sectional study	347 (men: 141, women: 206)	Global	Obesity was high among the participants without formal education.	OW: 44.7% OB: 15.3%	9
Kibria et al^48^	2016	Median age: 40 years	Nepal	Cross-sectional survey	12652 (men: 5283, women: 7369)	Both Asian and Global	Higher education level positively associated with OW/OB compared with people with no formal education.	Asian OB: 11.0% Global OB: 4.3% Asian OW: 26.4% Global OW: 18.2%	9
Gupta et al^43^	2016	Mean age: 45.2 years Range: 15-49 years	Nepal	Cross-sectional survey	6031 women	Asian	Women who attained primary education only had the highest prevalence of OW/OB.	OW: 23.7% OB: 11.6%	8
Poudyal et al^36^	2017		Nepal	Cross-sectional study	221 (men: 71.9%, women: 28.1)	Global	Literate participants had lower risk of OW/OB compared with illiterate participants (OR: 0.5, 95% CI: [0.3, 0.9]).	OW: 9.9% OB: 2.3%	7
Bista et al^35^	2019	Range: 15-69 years	Nepal	Cross-sectional survey	5593 (men: 47%, women: 53%)	Global	Participants with more than secondary level of education at lower risk of NCD risk factors compared with participants with less than primary education (ARR: 0.9, 95% CI: [0.8, 0.9])	OB/OW: 24.3%	10
Biswas et al^44^	2014	Range: 15-49 years	Bangladesh	Cross-sectional study	16624	Asian	Urban women were twice as likely (OR: 1.6, 95% CI: [1.4, 1.9]) to be overweight and three times more likely (OR: 3.0, 95% CI: [2.4, 3.7]) to be obese than rural women.	OW: 25.5% OB: 11.2%	9
Rawal et al^29^	2015	Range: ≥ 25 years	Bangladesh	Cross-sectional study	507	Global	Individuals with no formal education (*P* < .001) and those with only primary education (*P* = .01) were more likely to be overweight and obese.	OB/OW: 22.7%	9
Islam et al^45^	2016	Range: ≥ 18 years	Bangladesh	Cross-sectional study	450	Asian	Females had a higher obesity rate (30.4%) than males and were more than three times as likely to be overweight or obese (AOR: 3.37, 95% CI: [1.84, 6.15], *P* < .001).	OW: 40.5% OB: 28.0%	6
Gupta et al^46^	2017	Range: ≥ 18 years	Bangladesh	Cross-sectional study	12804	Asian	Those with high school education or above had 1.9 times higher odds of being overweight (AOR = 1.9, *P* = .033).	OW: 29.4% OB: 10.8%	8
Akseer et al^30^	2013	Range: 15-49 years	Afghanistan	Cross-sectional study	15558	Global	Higher education is linked to lower obesity rates, with women holding no formal education. Illiterate women were 1.5 times more likely to be obese (OR = 1.5, 95% CI: [1.1, 2.2]).	OW: 32.1% OB: 27.4%	8
Saeed et al^31^	2013	Range: 25-65 years Mean age: 38.8 years	Afghanistan	Cross-sectional study	1200	Global	There was a significant association between educational attainment and obesity, with illiterate individuals being nearly twice as likely to be obese compared with their literate counterparts (OR = 1.94, 95% CI: [1.41, 2.68]).	OW: 32.1% OB: 27.4%	8
Pengpid et al^32^	2018	Range: 18-69 years	Afghanistan	Cross-sectional study	3779	Global	Higher education levels (secondary or more) exhibited a lower prevalence of obesity (11.9%) compared with those with no formal education (20%) or primary education (15.4%). However, the difference was not statistically significant (*P* = .133).	OW: 25.5% OB: 17.2%	10
Rafique et al^33^	2013	Range: 18-69 years	Pakistan	Cross-sectional study	7366	Global	Obesity rates were highest among those with a postgraduate degree (20.2%, 95% CI: [14.1%, 26.2%]), compared with 11.7% (95% CI: [9.8%, 13.5%]) among those with no formal education.	OW: 26.3% OB: 14.9%	7
Pelzom et al^34^	2014	Range: 18-69 years	Bhutan	Cross-sectional study	2822	Global	Obesity was more prevalent among individuals with higher education levels. Those with secondary or higher education had a higher likelihood of being overweight compared with those with no formal education (aPR 1.46, 95% CI: [1.31, 1.63]).	OW/OB: 33.0%	9
Jayawardana et al^47^	2013	Range: 16-72 years	Sri Lanka	Cross-sectional study	2469	Asian	No significant association between educational attainment and obesity (*P* = .1246) was found.	OW: 31.8% OB: 12.3%	7
Somasundaram et al^49^	NA	Range: ≥ 18 years	Sri Lanka	Cross-sectional study	463	Both Asian and Global	The obesity prevalence was highest in the most educated group, and abdominal obesity was more prevalent in both the least (42%) and most educated groups (45%).	Asian OB: 31.2% Global OB: 15.8% Asian OW: 34.1% Global OW: 37.0%	7
Hashan et al^51^	2016	Range: 15-49 years	Maldives	Cross-sectional study	6634	Asian	There was no significant association between education level and obesity in women. The AOR for women with primary, secondary, and higher education were 1.0 (95% CI: [0.5, 2.1]), 0.8 (95% CI: [0.4, 1.8]), and 0.6 (95% CI: [0.3, 1.4]).	OW/OB: 63.0%	7

Abbreviations: aOR, adjusted odds ratio; ARR, adjusted relative risk; NCD, non-communicable diseases; OW, overweight; OB, obesity.

### Prevalence of OW/OB

Results indicated the varied prevalence of OW/OB across and within SA countries (Table 1). In India, overall prevalence of obesity ranged between 4.6% and 64.4%. In Nepal, over the same time period (2013-2019), the overall prevalence of OW/OB ranged from 12% to 24%. In Bangladesh (2014-2017) the overall prevalence of OW/OB ranged from 22% to 40%. Three studies from Afghanistan^30-32^ spanning 2013-2018 reported a prevalence of OB of 17% to 27%. A 2014 study from Pakistan reported the prevalence of OW as 26.3% and OB as 14.9%,^33^ while a study from Bhutan reported an overall prevalence of OW as 33% with no prevalence of obesity reported.^34^ Two studies from Sri Lanka (2013-2019) reported an overall prevalence of overweight ranging from 31.8% to 24.3% and obesity from 12.3% to 31.2%.^49,52^ A single 2016 study from the Maldives reported an overall prevalence of OW/OB as 63% among an all-female study population.^51^

### OW/OB and Educational Attainment

The relationship between educational attainment and OW/OB differed by country and study. The majority of studies from India, Bangladesh and Pakistan reported a direct relationship between OB/OW and educational attainment. Out of the 14 studies in the review from India,^20-26^,^38,40,42,50^ 11 reported a direct association between level of education and OW/OB, that is, higher the education higher the prevalence. Three studies reported either inverse^39^ or inverse U relationship^27,41^ between education and obesity such that OW/OB was clustered among the least educated or among those with median levels of education, respectively. Overall, 10 studies^20-26^,^40,42,50^ reported either an odds or prevalence ratio of OW/OB stratified by education. All ten studies described a stepwise increase in odds of being OW/OB with increasing education. In Bangladesh, two studies^44,46^ found that OB/OW prevalence increased with higher levels of education. One study^45^ reported an inverse relationship between educational attainment and OW/OB prevalence and one study^29^ reported highest levels of OW/OB in those with no education or only primary education. In Pakistan, OW/OB showed a stepwise linear increase with increasing education.^33^ The difference in the prevalence of obesity was significant between the two extremes of educational attainment (no formal schooling and postgraduate education). The same was not true for the prevalence of overweight in which there was an increased prevalence among those who were more educated, this difference did not achieve significance (overlapping 95% CI).

In Maldives, the association of education and OW/OB prevalence was indirect.^51^ The prevalence of OW/OB increased as educational level decreased with the highest proportion (82.9%) among women with only primary education (*P* < .001). Studies from Afghanistan and Nepal reported mixed results for the relationship between education and weight status. In Afghanistan, a study from 2013 reported a linear direct relationship between BMI and education levels.^30^ Another study from the same year reported an indirect relationship between education and obesity such that illiterate individuals were 1.94 times more likely to be obese.^31^

In Nepal two studies reported no association between educational attainment and OW/OB.^35,37^ One study showed a direct relationship between education and obesity,^28^ which is in contrast to the most recent study conducted in Nepal that showed inverse relationship.^54^ The remaining three studies^36,43,48^ showed an inverse U-shaped relationship between education and obesity such that the highest prevalence or odds of obesity was concentrated in either those with primary education or medium/secondary education. There was no association between education and weight reported in studies from Bhutan and Sri Lanka. In Bhutan, the prevalence of overweight ranged from 31-35% across different levels of educational attainment but was not significantly different. Adjusted Prevalence Ratios (APRs) showed a trend toward decreasing overweight with higher educational status, but this was not statistically significant.^34^

### OW/OB and Sex

Females were more likely to be OW/OB than males in all SA countries included in the review. In studies from India, among studies that stratified the prevalence of OW/OB by sex,^20-22^,^24,25,39,41,42^ all but one study reported a higher prevalence of OW/OB in males compared with females. This was consistent with studies from Nepal that reported prevalence of OW/OB by sex.^28^,^35-37^,^48^ While one study^28^ reported that females were less likely to be OW/OB, three studies^36,37,48^ reported that females had higher odds/prevalence of OW/OB, and one study^35^ reported no significant difference between males and females. Bangladesh showed a similar trend of females being significantly more likely to be overweight or obese compared with males.^29,46^ In the 2015 study,^29^ females were 3.37 times (aOR) more likely to be OB/OW. In 2017, females were 2.22 times as likely to be overweight or obese when compared with males.^46^

In the two studies reporting obesity prevalence by sex from Afghanistan, showed that females were significantly more likely to be obese than men.^31,32^ However, when comparing prevalence of OW, rates were similar in males and females.^31^ In the 2013 study, females were 2.94 times more likely to be obese than men (OR). Adjusted RR in the 2018 study reported females were 1.35 times more likely to be obese than men.^32^ While in Pakistan, there was no reported difference in the prevalence of overweight between males and females, but females were significantly more obese than males (17% versus 12%).^33^ In Bhutan, females were significantly more obese than men (20 vs 27%, aPR 1.46).^49^ Likewise, in Sri Lanka, females were reported to have higher prevalence of obesity (34.4 vs 24.2%) compared with males.^49^

### OW/OB, Sex, and Educational Attainment

Several studies reported prevalence of OW/OB stratified by education and sex^20,21,24,36^ and two studies reported OR of OW/OB by education and sex.^24,40^ In studies from India reporting prevalence, OW/OB was most common among females with primary/middle school education. The risk (aOR) of OW/OB in more educated men was higher than the risk for in more educated women. In Nepal, the highest prevalence of OW/OB among men were those with informal or no education, whereas the highest prevalence in females were among those with secondary education.^36^

### OW/OB and Area of Residence (Rural vs Urban)

Prevalence of obesity was higher in urban areas in India, Nepal, Bangladesh, Afghanistan, and Bhutan. The overall prevalence of OW/OB reported in urban studies from India ranged between 14% and 64% compared with 5% to 36% among rural participants.^24,25,38^ The prevalence of OW/OB in urban areas was twofold higher than among participants living rurally. This finding was consistent with the study conducted in Nepal, Bangladesh, and Afghanistan where higher incidences of OW/OB among urban populations was reported.^28,30,35^,^43-46^,^48^ Similarly, in Bhutan, living in an urban area led to a 1.36 increased risk of being overweight.^34^ Unlike other SA countries, the prevalence of OW/OB in rural and urban areas in the Maldives was similar (urban 61.4% vs rural 66.6%).

## Discussion

The prevalence of OW/OB appeared to be rising in four SA countries (Bangladesh, Maldives, Pakistan, Sri Lanka, and India), especially among females. In contrast, OW/OB seemed to be decreasing in the remaining three SA countries (Nepal, Bhutan, and Afghanistan). The association between educational attainment and OW/OB was reported as direct in India, Bangladesh, and Pakistan, indirect in Maldives, varied in Nepal and Afghanistan, and no association was found in studies from Bhutan and Sri Lanka.

In both Sri Lanka and Bangladesh, prevalence increased in both sexes, but more so in females.^29,49^ A recent study linked the notable rise in obesity prevalence over the last 14 years, particularly in females, to urban living conditions, increased income, and vulnerable age groups.^55^ Urban living conditions and higher income likely encourage sedentary lifestyles and results in higher purchases and consumption of processed foods. In addition, age-related metabolic changes and hormonal fluctuations further contribute to weight gain in females, acting as primary risk factors for the prevalence of OW/OB.^56^ Certain articles consider transitions in nutrition, dietary patterns coupled with socioeconomic factors as major contributing factors to the rising rates of obesity.^44^

Furthermore, in India, while there was a more substantial increase in prevalence in the overweight category for males, the increase in obesity among females was even more concerning.^20,22^ These findings highlight a public health concern, especially among the female population, necessitating urgent public health measures to tackle the problem. Numerous researchers view the shift from traditional to Western dietary patterns as a major factor in obesity.^57,58^ Some studies also suggest that varied body fat distribution between sexes may explain these differences.^59,60^ Women tend to accumulate fat in different body areas than men, potentially influencing obesity rates.^61^ The context was similar in Pakistan.^33^ A study in Pakistan emphasized the influence of sociocultural factors as well. Urban Pakistani women, often marrying young and remaining homebound afterward, were reported to be less physically active than their rural counterparts.^62^ This way of life renders them more susceptible to the prevalence of OW/OB.

Moreover, urban areas were associated with increased obesity rates in India, Nepal, Bangladesh, Afghanistan, and Bhutan, but not in the Maldives. Nonetheless, temporal trends in India and Bangladesh showed that this trend may be shifting, as rural areas exhibit a trend toward increasing OB/OW, while urban areas show a relative decline in OB/OW. This shift might indicate demographic shift in South Asia. Although wealth has long been linked with better access to food, and sedentary lifestyles, it appears to be changing to reflect trends seen in developed countries, where higher socioeconomic status is becoming a predictor of healthier diets and mor active lifestyles.^63^ For instance, a recent study conducted in India showed that individuals in higher quintiles generally consume less cereals and have increased consumption of fruits and vegetables.^64^

The association between education and OW/OB in SA countries seemed to evolve over time. A direct, positive association between higher levels of educational attainment and obesity has historically been common in various SA countries such as India, Sri Lanka, Bangladesh, and Nepal.^13^ While this study demonstrated a direct relationship between education and OW/OB in most studies conducted in India, recent work suggest that the association between higher education and OB/OW is diminishing.^20,42^ This was similar to observations in Sri Lanka. In Sri Lanka, previous work from 2005 to 2006 showed a positive association between education and OW/OB.^55^ This remained true in a 2013 study included in this review,^52^ but differences appear to be less significant in a more recent study published in 2019.^49^ In earlier studies, the relationship of education and OW/OB in Bangladesh has been previously shown to be positive.^65-67^ However, two other studies^29,45^ collecting data in 2015-2016 demonstrated the opposite association in which obesity was highest among those with less education. The relationship between obesity and education also appears to be changing in Nepal. In earlier studies, there was a clear stepwise increase in risk of obesity with increasing levels of education,^28^ which was consistent with previous data which showed that education level was associated with risk of metabolic syndrome.^68^ However, more recent data from 2019 did not show any difference between education groups.^35^

In Afghanistan, obesity and education has been historically indirectly associated.^69^ In this review, a study that collected data in 2013 showed that individuals who were illiterate were significantly more likely to be obese (OR = 1.94).^31^ However, in 2018, there was no significant difference in obesity by education representing a potential decoupling of education and obesity.^32^ In line with earlier research,^70^ this study identified one study showing that education indirectly influences obesity risk in the Maldives. Data from Bhutan and Pakistan is inadequate, rendering comparison to earlier time periods unfeasible. In this review, education showed a positive correlation with obesity both in Pakistan and Bhutan, although it was not significant.^33^ Although data varies across nations, there appears to be a shift away from a direct association between education and obesity that has historically defined the region. This could indicate a continuing economic shift in South Asia: as countries move toward more advanced stages of development, the relationship between education and obesity may start to mirror the indirect relationship between obesity and education observed in more developed nations.^71^

The relationship between education and OB/OW varied by sex in India and Nepal. In one study from India that reported OR by sex and education, education predicted OB/OW in both males and females.^40^ A second study reporting prevalence by education and sex, obesity prevalence was highest among males with primary and secondary education, and lowest among most educated males.^21^ Among females, OB/OW was highest among those with no or only primary education and lowest in those with graduate education and above. Furthermore, among females, education is a stronger determinant of obesity in rural communities than in urban communities.^24^ In men, there was little difference where education almost doubled the risk of obesity regardless of whether men lived in rural or urban communities. Taken together, this suggests that education as a gender-related variable may moderate OW/OB outcomes differently in males and females. For example, if rural communities have more traditional gender roles such as women’s prioritizing family preferences over their own nutritional needs^11^ and cultural norms that discourages women from engaging in outdoor physical activity due to safety concerns or modesty expectations,^72^ education may play a larger mediating role in determining OW/OB.^13^ Higher education levels increase women’s decision-making power in households, allowing them to prioritize their own health and nutrition rather than adhering strictly to traditional caregiving roles.^73^ Educated women are also more likely to engage in physical activity and social networks, countering restrictive norms that limit outdoor movement for females.^74^ Such gender roles significantly impact OW/OB patterns in SA countries.

This study was the first in the literature to examine sex and gender disaggregated data concerning obesity and education in South Asia, facilitating a better comprehension of the sex and gender-specific differences in the association between OW/OB and education. Furthermore, given that traditional gender roles limiting access to edutainment (e.g., cultural restrictions on media consumption for women reducing exposure to health-promoting sports-related content and confinement to passive entertainment)^75^ and prioritization of male education (e.g., families often investing more in boys’ education, leaving women with lower health literacy of nutrition and exercise contributing to unhealthy weight gain),^76^ significantly influence the sociocultural structure of South Asian communities, this study highlights the impact of certain cultural factors in health decisions. This study has some limitations. First, there was a wide variability in outcomes among studies and their definitions. This is particularly pertinent when studies have demonstrated a difference in OW/OB rates depending on the use of Asian or global BMI cutoffs. Applying South Asian-specific cutoffs might have increased OW/OB prevalence substantially that need to be taken into account for clinical diagnosis and intervention. Second, educational systems and their categorization varied by study and country, making cross-study comparison difficult and thus reducing generalizability. Third, the varied economic status of countries across South Asia might have diluted the association between educational attainment and OW/OB; however, such interaction between education and wealth is less evident in the context of LMICs, including South Asia.^77^ Finally, an unequal number of studies considered per country may have misrepresented study conclusion in this review.

## Conclusion

South Asian countries are undergoing substantial changes in economic, social, and political structure, which will persist in shaping how sex and gender interact with cardiovascular risk factors such as OW/OB. This study indicates that education may impact OW/OB differently in females and males. Females appeared to be at much higher risk for OW/OB across South Asia. While the methods by which increase of prevalence of OW/OB among females in South Asia is out of the scope of this review, this work indicates that efforts may need to be directed toward females. While earlier research typically indicated a steady direct correlation between education and OW/OB across most SA nations, our study proposes that this might not be accurate anymore. Due to the varying speeds of development among countries in this region, education’s function as a mediator of OW/OB varies by nation, indicating the necessity for evaluations tailored to each country.
